# Doing their damnedest to seek change: How group identity helps people with dementia confront public stigma and maintain purpose

**DOI:** 10.1177/1471301221997307

**Published:** 2021-02-18

**Authors:** Robert J Hagan, Sarah Campbell

**Affiliations:** Department of Social Care & Social Work, 5289 Manchester Metropolitan University, Manchester, England

**Keywords:** empowerment, consultation, group identity, stigma, purposefulness

## Abstract

Dominant messages about the capabilities of those with dementia post-diagnosis are often dehumanising and focused on mental declines. Additionally, carers for those with dementia are more likely to be involved in consultations and enquiries about the condition. This study helps to challenge stigmatising cultural messages by reporting upon the experiences of 13 adults diagnosed with early-stage dementia and how their involvement with empowerment groups in Northern Ireland has led to their involvement in consultations with policy makers and educational opportunities with the wider public. The study finds that this not only helps in challenging stereotypical ideas about dementia, as well as informing others, but also gives a sense of purpose to adults in their post-diagnosis lives. It is further noted that group identity helps give confidence and amplifies the voice of those who take part, allowing members to adopt a shared narrative and learn from each other.

## Introduction

A dementia diagnosis is sometimes associated with social withdrawal and inaction (Bosco et al., 2019; Gorska et al., 2018; Kinney et al., 2011). Diagnosed individuals may feel ashamed (Birt et al., 2017; Swaffer, 2014), lesser than others (Gove et al., 2016), helpless and useless (Ryan et al., 2009) and encumbered by grief and loss (Carone et al., 2016). Some fear they are useless (Langdon et al., 2007), and the stigma of the condition correlates with higher levels of anxiety and depression and lower levels of self-esteem and social participation (Harper et al., 2019).

Furthermore, cultural stereotypes of dementia influence those with the condition. Media representations portray ‘worst case’ scenarios to grab attention (Harper et al., 2019), leading to perceptions that a dementia diagnosis leads to a ‘social death’ – a loss of personhood and agency in terms of having any input into matters of relevance to those with dementia (Sweeting & Gilhooly, 1997; also Gilmour & Brannelly, 2010) – or a ‘living death’ (Birt et al., 2017). Dementia activist, Swaffer (2014) opines that these stereotypes are dehumanising and disrespectful, emerging from sensationalist storytelling that engages viewers and readers. Charities exacerbate attention-grabbing media messages to gain sympathy and support (Devlin et al., 2007). This leads to those with dementia being ignored or avoided in social situations (Gove et al., 2016). Negative public perceptions of dementia are so pervasive that those diagnosed believe them to be true and internalise for themselves (Harper et al., 2019), revealing how public stigma informs self-stigma (Nguyen & Li, 2020). Public stigma emerges from the reciprocal exchange of media-informed stereotypical knowledge around dementia publicly adopted, ‘othering’ those with the condition, seeing it as dangerous or piteous and leading to negative anxieties or avoidant behaviours. These messages include media hyperbole on how the condition is ‘worse than death’, a ‘bomb ready to explode’ and ‘the scourge of the 21^st^ century’ (Peel, 2014), reflecting a predominant focus upon the condition’s terminal phase, ignoring the voice of the person with dementia (Van Gorp & Vercruysse, 2012). As people with dementia receive and then believe these messages, self-stigma results, leading to self-imposed restrictions in terms of social engagement and agency. Yet, public stigma evolves from misleading and misconceived cultural narratives (Swaffer, 2014) that emphasise decline, degeneration and disappearance. This is summarised very effectively in this personal reflection from Richard Taylor in his foreword to Swaffer's (2016: 9) memoir:

‘We focus on the very end stage of the disease…. We act as if we’ve lost control of our lives, our dreams, our relationships. We begin to wait for the day our suffering ends and we will die, confused and alone…. The stigmas of dementia incubated between the ears of most everyone walking the earth’.

In contrast to this perception of hopelessness, research has found evidence of the retention of self post-diagnosis, though more tentatively in later stages (Caddell & Clare, 2010). This includes retention of capacity to make decisions, especially for those with mild or moderate dementia symptomology (Bosco et al., 2019; Smebye et al., 2012). People with dementia want to contribute to public discussions and consultations about the condition but stigmatising attitudes, including from practitioners, act as a barrier (Seetharaman & Chaudhury, 2020). As such, the lack of opportunities to engage both socially and civically is a cultural imposition (Beard & Fox, 2008), neglecting the benefits of continuing social engagement and participation (Górska et al., 2018). Being able to contribute, feel useful, make decisions and know others have expectations of you conserves self-esteem (Fetherstonhaugh et al., 2013).

Having some responsibility for shaping events and policies, as well as affecting or changing attitudes, is one hallmark of citizenship (Bartlett & O’Connor, 2010). However, those with dementia may experience the denial of rights and voice (Baldwin & Grearson, 2016). Citizenship, then, is the opposite of social death (Hughes, 2019) and can be transformative for those at risk of social exclusion. For those with dementia, this includes the right to be heard regarding concerns around service design, delivery and access (Buckner et al., 2019; Bartlett, 2016; Gilmour & Brannelly, 2010; Heward et al., 2017; ). In order to explore how people with dementia exercise this kind of citizenship, this article examines directly how 13 empowerment group members in Northern Ireland (NI) engage in both consultation and education and how these inform others of the realities of life post-diagnosis.

### Support groups as locations of purposefulness

To address potential social withdrawal and isolation, charities and advocates sometimes form support groups (Pratt et al., 2006). Group participation provides belonging, purpose, significance, security and continuity (Davies-Quarrell et al., 2010; Górska et al., 2018), with relationship building at the heart. Talking to group members with similar conditions counters self-stigma (Pierce et al., 2016), brings insight (Read et al., 2017) and delivers coping strategies (Mason et al., 2005). Gathering together gives those with dementia confidence to actively campaign for rights, raise awareness and contribute to debates (Birt et al., 2017). Therapeutic group work helps individuals overcome isolation, share knowledge and establish solidarity (Aminzadeh et al., 2007) and feel anchored, understood, consoled and accepted (Mason et al., 2005).

However, support groups may inadvertently create a group consciousness, which is excluding, narrow focused and forges individuals into diseased identities (Beard & Fox, 2008). Individuals with dementia may be reluctant to join social groups they feel are aimed predominantly at those with advanced dementia (Koehn et al., 2016) and wish to join organisations where dementia is not the primary focus (Carone et al., 2016).

Groups therefore can be both supportive and segregating, though adopting consciousness raising as a goal could enable group members to retain wider community connections. Whilst public stigma promotes the inability of those with dementia to express agency or initiate social action (Boyle, 2014; Beard & Fox, 2008), a diagnosis may actually serve as a catalyst to take action (Beard, 2004) and have a voice in decision-making (Beattie et al., 2004). The accumulation of first person accounts exploring living with dementia that have emerged over the last 15 years reflects this (Mitchell & Wharton, 2018; Swaffer, 2016; Taylor, 2007), with those who write memoirs inspired to counsel and educate others on how they should respond to people with dementia (Ryan et al., 2009). This literature has revealed how people with dementia are repositories of knowledge, able to bring insight to primary care practitioners (Minghella & Schneider, 2012), to speak at conferences and other meetings, and blog (Herron & Rosenberg, 2017; Read et al., 2017).

### Political context

Awareness raising is a key goal in NI’s dementia strategy (Department of Health Social Services & Publics Safety, 2011). Additionally, living well with dementia is central to the NI Executive’s policy for addressing the needs of those with dementia (Dementia Together NI/Northern Ireland Executive, 2016). This focuses on emphasising what individuals with dementia can rather than cannot do (Dementia Together NI/Northern Ireland Executive, 2016). In the United Kingdom and internationally, there are now numerous advocacy groups internationally with a focus on influencing policy and driving change (Seetharaman & Chaudhury, 2020). Opportunities for people with dementia’s social inclusion, civic participation and having choice and control over decision-making have been key messages in recent years (Department of Health, 2015; Bartlett, 2014).

Despite this, Buckner et al. (2019) reported that only around one-fifth of dementia-friendly communities actively involved people with dementia in their establishing, running and monitoring of activities and initiatives. Governmental goals, then, whilst including proactive initiatives to improve diagnosis processes, promote support, stimulate research and encourage more dementia-friendly communities, are ultimately more about what can be done for those with dementia rather than explicitly involving people with dementia in directives and policies (Department of Health, 2015). Surprisingly, the Department’s priorities as a result lack an explicit commitment to involve people with dementia in consultations.

### Hearing the voice

‘If I could feel useful to someone, it would be quite something’ (Alan, participant cited by Tanner, 2012: 301).

There is a dearth of research on how those diagnosed with dementia manage their new lives (Carone et al., 2016; Davies-Quarrell et al., 2010), and the contribution of people with dementia in citizenship roles is under-researched (Herron & Rosenberg, 2017; Bartlett, 2014). Whilst people with dementia are the experts in articulating their own experiences and discomforts (Swaffer, 2014), there has been doubt cast on the ability of people with dementia to contribute to social research studies (Menne & Whitlatch, 2007). It is carers, rather than those with dementia, who are more likely to be consulted in discussions relating to planning and services (Heward et al., 2017; Tanner, 2012), in the same way that medical practitioners have previously addressed their concerns primarily to carers (Gilmour & Brannelly, 2010; Sweeting & Gilhooly, 1997). The danger here is that carers’ needs may take precedence over the needs of those with dementia (Smebye et al., 2012). Increasingly, it is recognised that research on dementia excluding those with dementia is misrepresentative (Keady et al., 2017; Bartlett, 2014) with the views of carers insufficient in identifying the needs and perspectives of those with dementia (Kerkhoff et al., 2015).

A lack of understanding about the experiences of those with early-stage dementia partially reflects the under-resourcing of services for individuals at this point (Carone et al., 2016). This has rendered those with early-stage dementia susceptible to invisibility. Consulting those with dementia renders specific insights on identity and preferences of individuals (Menne & Whitlatch, 2007), as well as raises individuals’ self-esteem, self-confidence and sense of social inclusion (Tanner, 2012). Fetherstonhaugh et al. (2013) entitled their article ‘Being central to decision making means I am still here’, reflecting the value placed by people with dementia on being able to speak about matters important to them. Nevertheless, one caveat is that those with mild or moderate dementia do not speak for those with advanced symptomology (Tanner, 2012).

There is an emerging qualitative research base involving people with dementia as social citizens with the capacity and confidence to actively campaign and contribute to debates about their future care and support needs (Birt et al., 2017). For example, Oliver et al. (2020) describe the role of dementia-diagnosed individuals in a public and patient involvement forum, giving direction to dementia care within the United Kingdom, especially for those with young onset dementia. Both Rai et al. (2020) and Hassan et al. (2017) consulted with people with dementia regarding the use and appropriateness of user-friendly technologies and electronic devices. These studies not only allowed people with dementia the opportunity to provide instructive insights but also the very act of participation added to individual well-being and a feeling of value.

This current study builds on these contributions and investigates the under-researched area of people with dementia’s involvement in consultation and educational roles with policy makers and members of the public. The goals of the organisation under discussion align with guidance issued by the Department of Health Social Services & Public Safety (2011), which noted that the general public, public service workers and health and social care professionals all needed improved awareness of dementia.

## Methodology

Thirteen individuals who attend dementia empowerment groups in four locations in NI participated. As well as the opportunity to socialise with others with early-stage dementia, these groups arrange opportunities to educate members of the public and consult with policy makers about their experiences. The groups were developed by a member-led regional charity, originally founded by five individuals with dementia, who wanted to enable and empower others with a diagnosis to challenge stigmatised views of the condition and participate in consultations and lobbying. The co-chair of the organisation is one of the original founders, reflecting the charity’s desire to retain the control of those with dementia at the centre. Group membership is open-ended. Since their establishment in the last decade, they have produced TV and billboard advertisements about dementia, and members have recently contributed to writing guides about dementia and be involved in training those who work with people with dementia.^1^

The organisation also runs the aforementioned empowerment groups, which are facilitated by paid staff members. In this study, these facilitators aided in study recruitment. Sampling was purposive, limited to only those with a dementia diagnosis and who attended an empowerment group. Facilitators used their own skill and judgement to invite interested participants they felt would wish to participate and benefit from being involved. This ensured the researcher did not coerce any participants. Facilitators distributed an information sheet on the study prior to participation. Interviewees were aged between 48 and 80 years, with five under 60 years (See Table 1). Seven were women. This article is part of a wider investigation into the social integration of people with early-stage dementia.

**Table 1.** Profile of interviewees.

Name (Pseudonym)	Age (years)
Lorcan	74
Maolisa	74
Nuala	48
Oisin	55
Phelim	66
Quinn	74
Roisin	58
Stephen	80
Teresa	69
Ursula	78
Wilson	69
Yvonne	54
Zachary	55

### Ethical considerations

Whilst there may be ethical concerns around involving people with dementia directly in research (Tanner, 2012), the Mental Capacity (NI) Act 2016 directs capacity to be presumed (Harper et al., 2016). To safeguard care, group facilitators acted as gatekeepers, permitting only those in whom they were confident could consent to participate. Participants met the requirements of a functional test for capacity, with interviewees able to understand the research task, retain this information and weigh up the importance of this before making a decision (Lynch et al., 2017). In this study, all taking part signed consent forms, which the researcher read through with each participant, indicating their contribution was voluntary and could be withdrawn at any time. Participants were given specific information as to how their interviews would be recorded and used in potential journal articles. There was no remuneration.

### Procedure

The first author conducted 13 semi-structured interviews between June 2017 and April 2018 in the regular group locations or charity office in order to reduce potential anxiety. Interviews were recorded digitally, in full view of participants, and lasted between 25 min and one hour. An interview schedule was drawn up to cover topics exploring reasons for attending the empowerment group and changes to social lives since diagnosis. Interviewees were free to diverge from this and concentrate on matters of importance to them and were given pseudonyms to protect their identities. The research study gained ethical approval through the Ulster University Research Ethics Committee in December 2016 (reference REC/16/0102).

### Analysis

The researchers adopted an inductive grounded theory approach to examine matters important to interviewees that could then be subjected to theoretical elaboration (Haig, 1995). The approach allowed participants to stray from topics originally drafted, helping to lessen potential bias and prior knowledge (Beuscher & Grando, 2009; Wimpenny & Gass, 2000). This article reports on how participants often spoke, and with some pride, about the achievements gained from group membership.

Following interview transcription, the first author uploaded scripts to NVivo and undertook a line-by-line coding analysis to develop categories. To ensure interpretations of data were valid, rigorous and representative, the second author then reviewed interview scripts and coding. The two authors then agreed the final themes and content. Analysing data relating to the group’s focus on engagement with wider networks, four themes emerged. The first, tackling public stigma, relates to a wider principle underpinning reasons for engagement. The subsequent two themes relate to networks with whom the group has worked. The final theme reflects on benefits that accrue for group members.

## Findings

### Tackling public stigma

Respondents recognised public stigma. Once diagnosed, individuals feared being confined in stereotypical and stigmatised services.

‘It’s nice to get away from the memory thing, um, coffee thing that they have’ (Yvonne).

The charity overseeing the empowerment groups state that their first aim is to challenge stigma. This underpinning principle informs the fundamental purposes advising members as they engage with wider networks. Respondents were clearly aware that public perceptions of dementia were stigmatised and negative and were keen to challenge this.

‘We’re trying to do away with the stigma of dementia because it is, from what I’ve been told, [dementia is] going to be the biggest killer in the next 15/20 years’ (Lorcan).

Group members recognised how they had acquired commonly held misconceptions generated culturally or socially about dementia, which provoked uncomfortable thoughts and emotions that were self-stigmatising.

‘What I would hear about people before having dementia, God help them…, hope to God, you know, that never happen to me’ (Teresa).

‘Before I ever knew anything much about Alzheimer’s, I just thought you were done…, I didn’t… see that there was a future…. I really thought that anybody that had it were done…. That they couldn’t make decisions or couldn’t go to the bank or they couldn’t do all these things, you know, live a daily life and get messages or things like that…. People think you’re stupid, you know’ (Ursula).

‘I saw a couple of people were sort of saying, aw, that poor fella. I saw the expression’ (Wilson).

However, respondents identified that these public perceptions were skewed and misunderstood by others.

‘Life isn’t over, it’s really not. And that needs to get into the public as well’ (Zachary).

Thinking of how people with dementia may be perceived in community settings, Roisin outlined how her presentation to social work students challenged preconceived notions.

‘When they left the room, they all said… the stereotype typical person with dementia that they had come with in their mind, there was nobody in that room matched [that…] so they said it would mean in their training and then whenever they would go on to practice…, that if they learned anything it was you don’t presume…. They thought they were going to come into a room with everybody in their 80s sitting, nobody communicating at all really…, whereas they seen that wasn’t, definitely wasn’t the case’ (Roisin).

The interplay between public stigma and self-stigma was apparent in Teresa’s narrative, where she reflects on a lack of confidence about speaking in front of others. Her repetition three times of identifying how those with dementia are ‘okay’ reveals how she wrestles with preconceived notions that infer incapability.

‘I went to the… meeting up in Belfast…, it really set in then…, people sitting listening to people talking and I thought, well, they’re up there too…, they’re talking and, you know, they’ve dementia too. And they’re doing okay…. They’re all diagnosed the same as me. And they’re doing okay, even though I have it. They’re doing okay’ (Teresa).

Stigma, then, has a large part to play in encouraging group members to tackle misconceptions. The next two subsections outline how this is addressed.

### Wider network engagement 1: Confronting systems and challenging professionals

As the charity was respected for advocating on behalf of people with dementia, empowerment groups found themselves in the position of being asked to contribute to policy consultations. Group members keenly valued being able to address professionals and policy makers. Lorcan reflected a sense of frustration regarding his perception that changes were required.

‘What I want to see happen throughout all of the discussions that we’re having [is…] that there’s joined up thinking with the medical profession, with the hospitals and there isn’t, to be quite honest, but they’re getting better’ (Lorcan).

Group membership yielded access to those in power who were doing this thinking. Respondents appreciate the group’s role in being a respected pool of knowledge from which professionals and policy makers could draw.

‘If there’s changes being made in something, you know, you’re asked as somebody with dementia…, how could this be changed that would be of benefit to you’ (Roisin).

Some members saw the group, rather than themselves, as having power to drive change. In this way, some respondents distanced themselves from their contributions, instead conceptualising their involvement as representing the charity.

‘I think it’s great to see that they’re [i.e. the charity] starting to talk to the Trust or the Trust are starting to talk to [the charity] as well because I think the Trust think that they know what they’re doing and I don’t think they always do. And I think they think the last talk they had was an eye opener for them’ (Yvonne).

‘It’s the top people need to know what’s happening and they need to know that [the charity] have a real rule and a real ability to make change [though] I’m not convinced that that’s going to happen anytime soon’ (Lorcan).

Whilst these respondents submerged their involvement into a group response, Zachary recognised that group membership gave him access to tell his own story to powerful others.

‘I went to talk with [facilitator] to… [health] Trust members. I was very, very nervous but I’d wrote out a few things…. People were shocked that day in the meeting, you know what I mean? And I was happy enough… when I heard that people were shocked…. One of the guys is going to come to meet us here so I hope something really comes out of it…. I have certain goals that I want to see changed and I’ll do my damnedest to get those changed’ (Zachary).

Nevertheless, Zachary also recognised that promotion of the group was valued and meaningful in being representative of a broader network of voices.

‘I would like people to know what [the charity] are and what they do, you know, really in their face with it..., and that’s where I’d like to take it, into people’s faces and embarrass people into being aware of it and… change their own practices and views on dementia’ (Zachary).

Zachary recognised a power imbalance between his own position and those who could activate change. Group membership lessened this and granted access to those who were or could later be in powerful positions. Addressing those in training to be doctors, nurses, social workers or other allied health professionals was useful in instilling at an early stage a new narrative about dementia.

‘To me that is the future…, because they’re the people that are going to be caring for you when you do get to a much later state…. Hopefully it helps their training rather than it all being from a book or a lecturer…. You know, they’re meeting real people’ (Roisin).

Lorcan wanted changes in the local hospital, specifically in relation to signage, and there was some (cautious) sense in his narrative that the issue had been raised and was being addressed.

‘I’m told that they are getting better because if I’m going to the hospital now or go to A&E and I’ve trouble seeing the signs and things like that…, I’m told that they’re making a lot of headway in those areas’ (Lorcan).

The narrative is, however, hesitant and uncertain: Lorcan uses the disclaimer ‘I’m told…’ twice. There is a sense that there are processes for the group’s voice to be heard, but Lorcan is not convinced about how seriously the health trust receives this information. Power imbalances are reducing but not absent.

### Wider network engagement 2: Educating the public

Whilst group members’ contributions to consultations allowed them to advocate for change with policy makers, empowerment groups were equally keen to ensure that their own experiences and insights into dementia reached the wider public. Respondents spoke about addressing groups at colleges and universities, as well as wider and perhaps more unusual settings.

‘I’m going to the Tech [further education college] now on Monday to do a bit of training with children or young people’ (Yvonne).

‘I would have spoken, you know, at [farmers’] grasslands meetings and [to] breeders’ (Zachary).

‘What do you call those big factories where they do all the telecommunication things? …. Um… call centres…. They’re all listen really intently to me and us and I just felt I was doing good and I knew that those people would then be aware of dementia for the rest of their lives probably’ (Oisin).

Nuala revealed that their education role was not just about reflecting on personal experiences but recognising that they wanted to be better informed about relevant medical matters.

‘Until they’d asked, I didn’t realise what delirium was so I had to then research what delirium was and then realise, yeah, this is significant to me…, this could happen to me. And then write a speech and go and do the speech’ (Nuala).

The advocacy organisation also ran media campaigns locally on TV and billboards.

‘I done the advert…, me and my son… done the advert, all the posters that were all over, they were everywhere…. There was a TV advert. I done part of the first TV advert’ (Phelim).

Some members reported that opportunities could be challenging initially but found a way to push through discomfort to spread the news about dementia.

‘I’ve made some speeches… on what my life is like living with dementia… and that was pretty hard the first… but you sort of get a bit more confident’ (Oisin).

‘So I went over and walked a little with a woman and put my hand out and shook her hand. I said, “Hello, I’m Quinn and I have dementia.” Stopped dead. “Have you? My father died with dementia three years ago.” “Did he really?” So there we are, a 10-minute opening…. That’s a very good ice breaker, is shaking somebody’s hand. ‘Cos you can try and dodge them or they can try and dodge you but the hand, the handshake means more. I think it’s more sincere than a lot of words’ (Quinn).

Quinn’s use of touch is revealing in terms of not only demonstrating agency and a sense of control but also in exuding warmth and confronting the public stigma narrative of dementia being dangerous. Equipped with new insights and confidence, Teresa became an active citizen, challenging shops, businesses and health providers about ensuring accessibility.

‘No matter where I go, if I go into the dentist, are you dementia friendly…? I was in Marks and Spencer there a few weeks ago…, and I’ve asked them ones about are you dementia friendly?’ (Teresa)

Zachary recognised that different contexts may need varied approaches and there were communities still to reach.

‘[In rural parts of NI] people aren’t that open really and farming communities aren’t that open…, and I think I would like to see [the charity] overcoming that, just with getting out there… into the public and getting into shops and businesses and even healthwise…. Really making [the] public aware of [the charity] and… that people aren’t that different really’ (Zachary).

### Purposefulness

Undertaking this work accrued personal benefits, allowing group members to maintain a sense of purposefulness beyond their diagnosis.

‘You feel as if you’re doing something positive as well. You still have some worth, you still have some purpose’ (Roisin).

‘We’re doing… things like that so you’re treated as an adult and doing adult things, whereas you don’t want to be mothered or wrapped in cotton wool or anything else’ (Yvonne).

‘Even though it’s hard to do, it’s keeping your mind active’ (Nuala).

With this, there was a desire that there was meaning to the purpose, important messages to communicate.

‘I want to make awareness, I really, really want to speak out about dementia’ (Teresa).

‘I’m going to tell everybody that needs to know that I have dementia…, I have dementia but I’m fine. That’s the two words I said. I have dementia but I’m fine’ (Wilson).

‘There’s not really much point in being in [the empowerment group] unless you’re able to… do something’ (Nuala).

It was recognised that the group identity was important in achieving access to spaces perceived as inaccessible to individuals.

‘What opportunities would I get as me? None, ‘cos I can’t go along to Queen’s [University Belfast] and say, “I want to speak to a group of students.” They’d [say], “Aye, catch yourself on”’ (Roisin).

However, becoming known through group involvement as someone who speaks out about dementia leads to opportunities to discuss this in other areas of individuals’ social lives.

‘I was heavily involved in the GAA [Gaelic Athletic Association] and… people would come to me and would say, “I know so and so has dementia but he doesn’t admit it. Can you do anything about it?”’ (Lorcan).

The group activities facilitated busyness and at least partially compensated for missing lost employment.

‘I loved my job, every day was so different and you just were meeting so many people and you were out and about sort of all the time. I really did. I loved it. So for me it was quite a blow…. Whenever you’re working, it’s lovely to have a couple of weeks when you have nothing in your diary…, when you know you’re off and that’s it but for that to be every day, every week, every month I think would be soul destroying’ (Roisin).

‘Will you go and meet, saying things like this here? Will you go there? .… Gradually I was just going to one or two but I’m going to more and more now because it’s… something to look forward to. It’s something that takes your mind off the dementia and if there’s other people with the dementia that you can have a good craic’ (Phelim).

A further compensation was that the organisation provided opportunities to rekindle skills and practices prior to diagnosis.

‘I’m going to do like a charity thing down there sometime near Christmas in the future and I would have been good at them things before’ (Teresa).

Finally, Phelim also identified that certain knowledge and support accrued from being part of a wider network.

‘If you think you have it, seek help. I didn’t think there was help out there but there is, there’s loads of help out there. You just need to take that step and find out’ (Phelim).

## Discussion

This article has presented how those with early-stage dementia still have a voice post-diagnosis and achieve purpose through opportunities to share experiences, insights and knowledge with the public and policy makers. As noted earlier, literature on groups for those with dementia has sometimes described these as segregating, excluding and stigmatising. Whilst the empowerment groups here are exclusive to those with dementia, they never presume to be the only outlet for members’ socialisation. Rather, groups proactively encourage a group identity that engages with wider networks. The charity under discussion is just one part of a wider global movement of activist organisations comprising those with dementia and promoting advocacy and autonomy, such as DEEP in the United Kingdom (dementiavoices.org.uk) and Dementia Alliance International further afield (dementiaallianceinternational.org).

Individuals both gain and retain skills through membership. Zachary’s narrative revealed that the group was an effective conduit through which he could speak out about perceived deficiencies within the system. The group’s profile, bolstered by formal guidance in NI that policy makers listen to those with dementia, enabled Zachary to express his own ideas to previously inaccessible powerful voices. As such, contributions made through seeking change from health and social care policy makers reveal that the group demonstrates aspects of meta-citizenship, whilst the narratives recounting how individuals support and encourage each other exemplifies micro-citizenship (Baldwin & Greaser, 2016). Other group members conceptualised their own contributions less as reflecting their own voices but as being submerged into a wider group narrative: it was not they alone but the charity that was advocating for change. Nevertheless, group membership helped awaken and embolden existing passions and concerns. Roisin revealed that without access to a group identity, she would lack opportunity to voice concerns. More than this, being part of the group legitimised narratives as they became part of a louder voice on understanding dementia.

Crowd theory helps explain the desire to submerge into an organisation’s message, where members may lose a sense of individuality but gain power due to a shared identity (Reicher, 2001). Crowds do not nullify keenly held beliefs but rather amplify individuals’ values and give confidence and power to fight collectively for change (Neville & Reicher, 2018). They also lead individuals to feel they are not exposed but the group’s voice helps share accountability, with boldness increasing as a result (Postmes & Spears, 1998). Group members felt heartened by seeing others step out and speak up, and this was sometimes required before individuals decided to contribute, as group member Maolisa reflects:

‘I’ve learned a lot from Lorcan…. I heard him first of all on the radio and… when I listened to him it made me think… about people having dementia, not necessarily me…. When I listened to Lorcan… I thought definitely can’t be as bad as I making it out to be. So that sort of spurred me on.’

This and wider findings reveal how members inspire, inform and even politicise each other. This may come at the cost of groups largely representing only the viewpoint of their most powerful or vocal members. The empowerment group is not free from bias nor does it have an absence of values, and without critical reflection, there is potential here too for group members to readily and expediently assent to dominant views.

Nevertheless, if such groups do not exist, there is a precarity for those with dementia who have anxieties about care matters. Individually, their voices sink in an ocean of opinion, but once part of an empowerment group, access to the powerful is opened. This is a double-edge sword: the concept of equal citizenship is defined not by how individuals contribute but the extent to which the powerful listen to them (Bartlett & O’Connor, 2010). Whilst involvement in consultations on service delivery helps maintain dignity and self-respect (Lorentzon & Bryan, 2007), the empowerment group may inadvertently be subjected to ‘tokenism’ (Seetharaman & Chaudhury, 2020) by public figures, who conduct consultations as lip service to following departmental guidelines in terms of listening exercises, allowing (potentially insecure) leaders and policy makers to retain power (Neville & Reicher, 2018). Citizenship is always activated in relation to others, who have power to help, hinder or sustain (Baldwin & Grearson, 2016). The empowerment group’s very involvement is at the behest of the public organisation who offers (or could withhold) the opportunity (Seetharaman & Chaudhury, 2020; Birt et al., 2017), so there is an inherent power imbalance within consultations. Challenging public figures can be troublesome for a charity, which relies on funding and support from other sources for survival and exists only inasmuch as their importance is valued by those holding the purse strings. Whilst members negotiate this tightrope, the narratives still remain striking due to the clear passion individuals have to continue to say uncomfortable things.

**Figure 1.**
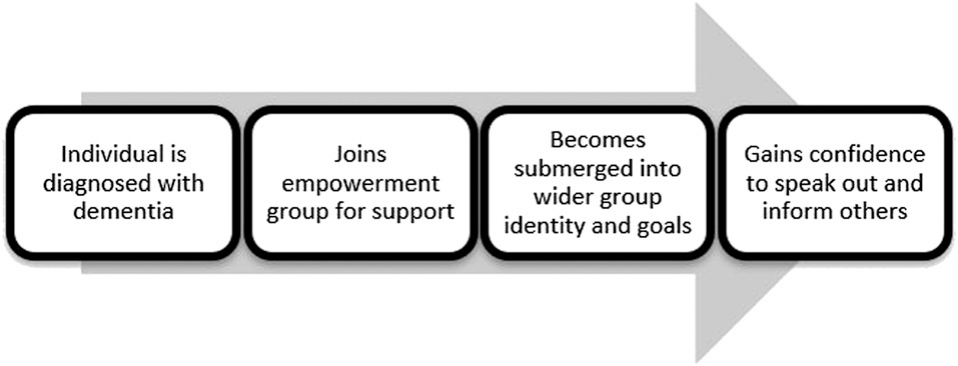
Hypothetical model describing the process to becoming a dementia advocate.

With these caveats in mind, Figure 1 summarises the process involved for group members, following a dementia diagnosis to the point where they feel ready to be involved in opportunities for education or consultation. This process takes time for individuals, reflecting not only the accumulation of experience post-diagnosis but also a gradual recognition of group identity and values, which broadly align with their own perceptions. At this point, and with support from group facilitators and other members, will they allow their voice to be heard?

Contributions made by individuals with the public also help challenge the accrual of public stigma around dementia. The broader societal view remains that the individual with dementia is in a liminal state transitioning from life to death (Birt et al., 2017; Sweeting & Gilhooly, 1997); the implication being that inherent personhood is lacking or compromised. Roisin’s account of speaking to social work students not only helped students recognise her capability but also overcomes their preconceptions informed by the broader and more powerful cultural perception of dementia. Roisin challenges the idea that the person with dementia is, at best, someone who should be protected and have an advocate or carer make decisions on their behalf (Fetherstonhaugh et al., 2013) and, at worst, incapable and subaltern, not worthy of being listened to (Gilmour & Branelly, 2010). The very nature of engagement was sufficiently powerful to overcome the influence of stigmatising media messages. As long as these encounters continue, the opportunity exists for public stigma to be challenged and reconfigured.

When group members, such as Maolisa’s reflection on Lorcan’s radio interview, see each other participate in educative and consultative events, they not only gain confidence about doing this for themselves but also, like the social work students, they engage in a self-reflective exercise where they realise that they may have adopted public stigma messages and effectively self-stigmatised. In the case of Yvonne, a former nurse trainer, obliged to retire following her dementia diagnosis, her own self-confidence was badly affected. However, her involvement with the empowerment group and, particularly, seeing other members deliver sessions led to her desire to revisit old skills.

‘I don’t want to do anything the first session and I want to see the couple of guys who’ve been in, what they do and see how they do it and then, so many months down the line, put my stamp on it and see how to go forward. But at least I have that option of taking forward the skills I do… have.’

This educative and consultative work also aligns with the stated goals of dementia-friendly communities, which should comprise galvanised individuals who instigate change and promote community action (Bartlett, 2016). The very presence of diagnosed individuals yields benefits in countering messages that the public struggle with how to respond to those with dementia and are fearful or anxious (Devlin et al., 2007). Opportunities afforded the empowerment groups, then, are not just to the members but also to the public and policy makers. If this is lost, the ability for individuals to raise issues of concern with those in power is severely compromised.

Finally, this article has added to the growing body of studies directly reporting the views and opinions of those with dementia. When research ignores these voices, the understanding and theory accrued is built on giving greater weight to the concerns of carers and professionals, as well as media scares that are more likely to present messages that heighten fear, anxiety and narratives of helplessness (Peel, 2014). As such, continuing to engage with people with dementia is vital in ensuring a more rounded and fairer picture represents life post-diagnosis.

### Limitations

The study reports on the views of 13 individuals in four different areas of NI, and there may be challenges replicating findings to other contexts. The interviews represent those with early-stage dementia and may not adequately capture the insights and distinct needs of those diagnosed longer and for whom the condition is more pronounced. A future direction for further research could be how the voice of those whose dementia has progressed is included. Additionally, it would be useful to gain insight into how empowerment groups like these, and the charities who facilitate such groups, navigate the difficult waters of members’ decreasing capacity and confidence and what directions or guidance is offered during this.

The discussion has identified the potentially troublesome nature of the charity’s involvement in public consultations. A further limitation is that this study has not attempted to measure or capture the effectiveness of this or to what extent change has been activated beyond anecdotal evidence from the participants themselves, for example, Lorcan’s recounting of (potentially) improved signage at his local hospital.

## Conclusion

This study has reported on the valuable insights that people with dementia bring to bear on their own situation. It has argued that the confidence instilled by virtue of group identity leads to visible and meaningful contributions and helps challenge ideas that stigmatise not only the public but also those with dementia themselves. Although a group could be compromised by the negative dynamics of groupthink or used as lip service, the potential losses in terms of knowledge of and sensitivity to a post-diagnosis life incurred by a group’s demise would be debilitating not just to those with dementia but for those who promote and respect inclusive communities. The individuals benefit from their contributions, helping them maintain a sense of continuity and having not only opportunities to socialise but also a purpose in which they take pride.

## Note

1 In order to protect anonymity, references for the material in this paragraph have been withheld but can be supplied upon request to the first author.
